# Acceptance of temporal artery thermometry by Nigerian mothers: a comparison with the traditional methods

**DOI:** 10.11604/pamj.2014.19.263.2899

**Published:** 2014-11-11

**Authors:** Odinaka Kelechi, Edelu Benedict, Emeka Nwolisa, Amamilo Ifeyinwa, Okolo Seline

**Affiliations:** 1 Department of Paediatrics, Federal medical Centre, Owerri, Imo State Nigeria; 2 Department of Paediatrics, University of Nigeria Teaching Hospital, Enugu State, Nigeria; 3 University of Jos Teaching Hospital, Plateau State, Nigeria

**Keywords:** Thermometry, mercury-in-glass thermometer, infrared temporal artery thermometer

## Abstract

**Introduction:**

Temporal artery thermometry may be viewed as a suitable alternative to the traditional thermometry because of its safety and time efficiency. However, it is yet to gain wide acceptance in African settings because it is relatively new. The aim of this study was to compare the choices of Nigerian mothers between the traditional methods (axillary and rectal thermometry) and the temporal artery thermometry.

**Methods:**

Rectal, axillary and forehead temperatures were measured in 113 children using rectal and axillary mercury in glass thermometers and infrared temporal artery thermometer respectively. The thermometry method preferred by each mother and the reason(s) were documented using a semi structured questionnaire. The data was analysed using SPSS version 19.

**Results:**

The highest number of mothers 44(38.9%) preferred the axillary route while 42(37.2%) and 27(23.9%) preferred the temporal and rectal routes respectively. Temporal artery thermometry was the most popular among the mothers with tertiary education 27(39.7%), whereas axillary thermometry was most preferred among mothers with primary and secondary education, although this difference was not statistically significant (χ^2^=0.62,p = 0.96). Mothers 27(33.9%) who preferred rectal thermometry did so because they felt that since the thermometer is inserted inside the body, it will detect fever better.

**Conclusion:**

Nigerian mothers do not have any particular thermometry preference between the temporal artery thermometry and the traditional methods, so medical personnel in our environment may resort to any method that is convenient, accurate, fast and cost effective.

## Introduction

Fever is one of the commonest reasons for presentation to the health facilities, especially in the under- 5 age group. It is a good indicator of morbidity in children. Many care givers show a lot of concern about the effect of fever on their children [1].

Temperature measurement in children in most health facilities in our environment is usually done using the axillary mercury in glass or digital thermometer. The axillary method is simple, safe, hygienic and easy to use but some authors have considered it time wasting [2, 3]. The rectal route which several authors have considered the gold standard for temperature measurement is still used in some centres in Nigeria, especially in neonates. The rectal route is considered the most practical route that closely reflects the core temperature [4]. However, some caregivers have expressed their discomfort and resentment about the procedure [5]. In addition, it requires the maintenance of good hygiene and the fear of perforation or breakage of thermometer adds to the drawbacks with the use of rectal thermometry.

Technological advancement has made available several other thermometry methods such as the temporal artery scanner which has simplified the process of temperature measurement. The instrument utilizes infrared technology to detect core body temperature emitted from the skin surface through the temporal artery. Although the device is widely utilized in most developed countries, its accuracy is still not established with several studies giving conflicting results [6–8]. In separate studies by Titus et al [6] and Siberry et al [7], the device was found to be an effective screening tool for fever, whereas, Greenes and Fleisher [8] reported limited sensitivity even though it was better tolerated than rectal thermometer.

In our environment, the device is still new and it is not clear if the mothers and caregivers will prefer its use to the axillary and rectal methods they are used to.

This study was aimed at comparing the choices of Nigerian mothers between temporal artery thermometry and the traditional methods (axillary and rectal thermometry)

## Methods

This cross sectional study was undertaken at the paediatric department of Federal Medical Centre Owerri, Nigeria over a six month period from July to December 2012. Children under 5 years of age whose mothers consented were recruited from the children outpatient and emergency paediatric units. Ethical approval was obtained from the hospital research and ethics committee before commencement.

Eligible children had their temperature measured from the forehead, axilla and rectum using the temporal artery thermometer (TAT-2000C Exergen USA) and standard mercury in glass axillary and rectal thermometers respectively. Children who were judged to be critically ill were excluded from the study.

The axillary thermometer was first inserted into the axilla and left for 5 minutes thereafter, the rectal thermometer was lubricated and inserted into a depth of 3 centimetres inside the anus and left for 3 minutes. The temporal artery temperature was measured just after removing the rectal thermometer by placing the temporal artery thermometer probe on the centre of the forehead while pressing and holding the scan button and gently slid towards the right to the hairline while keeping the sensor flat and in contact with the skin for the entire scan. The scan button was released once the probe gets to the hairline. This process took about 3 seconds and the temperature on the display was read.

Thereafter, the mothers were asked to identify their preferred method and the reason(s) for their choice. No options were given to them on the reasons for their choices. Each mother was allowed to express herself and say why she preferred the method chosen. These and other information were documented a semi-structured questionnaire. Their reasons were categorized and with other data analyzed using the SPSS version 19 (IBM Inc. Chicago Illinois, USA, 2010).

## Results

A total of 113 mother-child pairs were recruited for the study. The children aged from 2 days to 59 months with a mean age of 9.6 months. There were 60 males and 53 females giving a male: female ratio of 1.1: 1. The mothers were aged from 19 to 46 years with a mean age of 27.4 ± 5.3years and a median age of 27 years. All the 113 mothers had some form of formal education; 60.2% had tertiary education, 33.6% had secondary education while the rest (6.2%) had only primary education.

The highest number of mothers 44(38.9%) preferred the axillary route while the rectal route 27(23.9%) was the least preferred as shown in Table 1. An analysis of the choices of thermometry among the different educational levels showed that temporal artery thermometry 27(39.7%)was the most popular among the mothers with tertiary education, whereas axillary thermometry was most preferred among mothers with primary and secondary education, but these differences were not significant. Table 2 shows the comparison. Cramer's V test for symmetrical measures has a value of 0.053, which showed that only about 5% of the choice of thermometry was accounted for by educational qualification.

**Table 1:** Mothers’ choice of thermometry

Measuring site	Frequency	percentage
**Temporary artery**	42	37.2
**Axillary**	44	38.9
**Rectal**	27	23.9
**Total**	113	100.0

(χ^2^ = 4.58, p= 0.101)

**Table 2:** Comparison of choice of thermometry with mothers’ highest educational qualification (HEQ)

	Thermometry choice		
HEQ	Temporal n(%)	Axillary n (%)	Rectaln (%)
**Primary (n =7)**	2 (28.6)	3 (42.9)	2 (28.6)
**Secondary (n =38)**	13 (34.2)	16 (42.1)	9 (23.7)
**Tertiary (n = 68)**	27 (39.7)	25 (36.8)	16 (23.5)
	42 (37.2)	44 (38.9)	27 (23.9)

(χ^2^ = 0.62, p = 0.96)

All the mothers 27(23.9%) who preferred the rectal thermometry did so because they felt that since the thermometer is inserted inside the body, it will detect fever better, while majority 24(57.1%) of the mothers that preferred the temporal artery thermometry did so because it was fast. Table 3 and Table 4 summarizes the reasons.

**Table 3:** Reason for choice of thermometry

Category	Reason
**A**	Fast
**B**	Will detect fever better since it's inserted into the body
**C**	More familiar method
**D**	Same site used in palpation for fever
**E**	Easy and Convenient
**F**	Fast and Easy
**G**	Others

**Table 4:** Reason for choice of thermometry

Site		A	B	C	D	E	F	G	Total
**Temporal**	n(%)	24(57.1)	0(0.0)	0(0.0)	3(7.1)	6(14.3)	7(16.7)	2(4.8)	42(100.0)
**Axillary**	n(%)	0(0.0)	0(0.0)	25(56.8)	0(0.0)	17(38.6)	2(4.6)	0(0.0)	44(100.0)
**Rectal**	n(%)	0(0.0)	27(100)	0(0.0)	0(0.0)	0(0.0)	0(0.0)	0(0.0)	27(100.0)

## Discussion

Temperature measurement in children is an everyday procedure in hospitals, especially for children presenting with fever. The method used may vary from facility to facility but in most health facilities in Nigeria, axillary thermometry is mostly employed although, rectal thermometry is also employed especially in neonates. Many Paediatricians in response to the ever increasing innovations in the temperature measuring methods as well as reports on the drawbacks of the traditional methods continue to change their thermometry methods without recourse to the mothers and caregivers [3–5, 9].

This study shows that Nigerian mothers do not have particular preference of thermometry, with 42% and 44% of the women showing preference for temporal artery and axillary thermometry respectively. This contrasts with a study conducted among Latin American parents where axillary thermometry was the preferred method while the newer tympanic thermometry was preferred by just 1%. [10] In a study in Turkey, 91.2% of mothers preferred measuring their child's temperature form the axilla [11].

Although the newer method tended to be more popular among the more educated mothers, educational qualification did not show a significant relationship with choice of thermometry (χ^2^ = 0.62, p = 0.96). Exergen, the manufacturers of temporal artery scanner had described the thermometer as “a kinder and gentler way to take temperature”, [12] thus, one would have expected a greater preference of the thermometer for that reason. However, most mothers who preferred the newer temporal artery thermometry did so because it took less time. This gives credence to what some authors had previously reported as one of the reasons why the traditional methods, especially the axillary thermometry which take as much as 5 -7 minutes to complete should be replaced. [2, 13, 14] The use of palpation to detect fever, although unreliable is still a very widely practiced method in our environment. [15, 16] Some mothers believe the temporal artery thermometry will detect fever better since it's employed at the same site as that used for palpation by many mothers.

Mothers who preferred the axillary thermometry probably represent the group that are resistant to change which is not unusual in any process one finds comfortable. A good number of mothers consider it easy and convenient probably because they can utilize the method in the homes with less difficulty.

All mothers who preferred rectal thermometry considered it as the method that will best detect fever because of the position of the thermometer. This would suggest that many mothers do not mind the idea of inserting thermometer into the rectum provided the fever is detected.

Since the rectal thermometry is still considered the most accurate and practicable method for use in the hospital setting, it may be necessary to utilize it where the logistics permit. The rush to introduce newer methods may result in the erosion of the confidence of some mothers who prefer the traditional thermometry methods. Duncan keeley, in a review article recommended the use of rectal thermometry over axillary because it is faster, more accurate and less disturbing to the infant.

## Conclusion

Nigerian mothers do not have any particular thermometry preference between the temporal artery thermometry and the traditional methods, so medical personnel in our environment may resort to any method that is convenient, accurate, fast and cost effective.

## References

[1] Crocetti M, Moghbeli N, Serwint J (2001). Fever phobia revisited: Have parental misconception about fever changed in 20 years?. Pediatrics..

[2] Ng DKK, Lam JCY, Chow KW (2002). Childhood fever revisited. HKMJ..

[3] Osinusi K, Njinyam MN (1997). Comparison of body temperatures taken at different sites and the reliability of axillary temperature in screening for fever. Afr J Med Sci..

[4] El-Radhi AS, Barry W (2006). Thermometry in paediatric practice. Arch Dis Child..

[5] Kai J (1993). Parents’ perceptions of taking babies’ rectal temperature. BMJ..

[6] Titus OM, Hulsey T, Heckman J, Losek JD (2009). Temporal artery thermometer utilization in pediatric emergency care. Clin Pediatr..

[7] Siberry GK, Diener-west M, Schappell E, Karron RA (2002). Comparison of Temple temperatures with rectal temperatures in children under 2 years of age. Clin Pediatr..

[8] Greenes DS, Fleisher GR (2001). Accuracy of a noninvasive temporal artery thermometer for use in infants. Arch Pediatr Adolesc Med..

[9] Craig JV, Lancaster GA, Taylor S, Williamson PR, Smyth RL (2002). Infrared ear thermometry compared with rectal thermometry in children: A systematic review. Lancet..

[10] Crocetti M, Sabath B, Cranmer L, Gubser S, Dooler D (2009). Knowledge and management of fever among Latino parents. Clin Pediatr..

[11] Arica SG, Arica V, Onur H, Gallayzar S, Daa H, Obut (2012). Knowledge, attitude and response of mothers about fever in their children. Emerg Med J.

[12] Exergen Corporation Temporal scanner: A kinder gentler way to take temperature. Reference manual.

[13] Van Staaij BK, Rovers MM, Schilder AGM, Hoes AW (2003). Accuracy and feasibility of daily infrared tympanic membrane measurements in children. Int J Pediatr Otorhinolaryngol..

[14] Chaturvedi D, Vilhekar KY, Chaturvedi P, Bharambe MS (2003). Reliability of perception of fever by touch. Indian J Paediatr..

[15] Akinbami FO, Orimadegun AE, Tongo OO, Okafor OO, Akinyinka OO (2010). Detection of fever in children emergency care: comparison of tactile and rectal temperatures in children. BMC Research Notes..

[16] Keeley D (1992). Taking infants’ temperatures: forget the axilla the rectum is better. BMJ..

